# Pilot Study of AI-Assisted ANA Immunofluorescence Reading—Comparison with Classical Visual Interpretation

**DOI:** 10.3390/jcm14196924

**Published:** 2025-09-30

**Authors:** Sarah Mayr, Margit Dollinger, Boris Ehrenstein, Florian Günther, Olga Krammer, Antonia Schuster, Thomas Büttner, Rico Hiemann, Peter Schierack, Dirk Roggenbuck, Martin Fleck

**Affiliations:** 1 Department of Rheumatology and Clinical Immunology, Asklepios Medical Center Bad Abbach, 93077 Bad Abbach, Germany; 2 Medipan GmbH, 15827 Dahlewitz, Germany; 3 Institute of Biotechnology, Faculty Environment and Natural Sciences, Brandenburg University of Technology Cottbus-Senftenberg, 03046 Cottbus, Germany; 4 Department of Internal Medicine I, University Medical Center Regensburg, 93042 Regensburg, Germany

**Keywords:** antinuclear antibodies (ANA) immunofluorescence testing 2, artificial intelligence 3 diagnostic for rheumatic diseases

## Abstract

**Background:** Antinuclear antibodies (ANAs) play a crucial role in diagnosing systemic autoimmune rheumatic diseases, particularly systemic lupus erythematosus. The recommended standard for ANA detection is indirect immunofluorescence testing (IIFT) using human epithelial (HEp-2) cells. Since visual interpretation (VI) of IIFT images is time-consuming and labor-intensive, research is focusing on automated interpretation systems that use artificial intelligence (AI). **Methods:** Consecutive serum samples (number of sera = 143) from routine clinical care were collected from patients visiting our tertiary rheumatology center. ANA were detected by IIFT with visual interpretation and compared with IIFT using the AI-based interpretation system akiron^®^ NEO (Medipan, 15827 Blankenfelde-Mahlow, Germany). ANA titer levels and patterns were analyzed according to the Competent Level of the International Consensus on ANA Pattern classification. **Results:** Agreement of positive/negative ANA discrimination between AI-aided and VI-IIFT at the recommended cut-off of 80 was good (Cohen’s kappa [κ] 0.69) but significantly different (McNemar test, *p* < 0.0001). At a cut-off of ≥1/80, the agreement was improved (κ 0.76) and the difference between both methods was non-significant (*p* = 1.0000). The ANA pattern recognition agreement between both approaches was moderate (κ = 0.54). The direct comparison using only the akiron^®^ NEO HEp-2 cell ANA assay revealed a good agreement (0.67), which improved to very good (κ = 0.80) when differences between ANA patterns anti-cell (AC)4/5 and AC2 were neglected. Notably, titer levels in the automated evaluations were frequently assessed at higher values than in the gold standard interpretation. **Conclusions:** Our study demonstrates a good agreement for positive/negative ANA discrimination. ANA pattern recognition by AI-aided interpretation showed moderate to very good agreement with VI. Further research and algorithm refinement (e.g., improved pattern recognition and titer calibration) are necessary to support its future implementation as a reliable screening method.

## 1. Introduction

Antinuclear antibodies (ANA), also referred to as autoantibodies to cellular antigens, are key laboratory markers used to screen for and support the diagnosis of systemic autoimmune rheumatic diseases (SARD) [1,2]. Indirect immunofluorescence testing (IIFT) on Human Epithelial-2 (HEp-2) cells is currently recommended for ANA detection due to its high sensitivity and broad antigen coverage. ANA testing plays a crucial role in the diagnosis and classification of connective tissue diseases, particularly systemic lupus erythematosus (SLE). According to the 2019 EULAR/ACR classification criteria for SLE, a positive ANA test is defined as a mandatory entry criterion [3,4,5].

In the ANA-IIFT, patient serum is incubated in serial dilutions with fixed HEp-2 cells on a microscope slide. Bound immunoglobulin G (IgG) antibodies are detected using fluorescein-labeled anti-human immunoglobulins and visualized under a fluorescence microscope. The resulting staining patterns provide valuable information on the presence of specific autoantibodies and guide further reflex testing for their confirmation. According to the International Consensus on ANA Patterns (ICAPs), laboratories should report both the ANA titer and the staining pattern to support standardization and enable appropriate reflex testing for specific autoantibodies [6,7].

However, the visual interpretation of ANA patterns by IIFT (VI-IIFT) is highly dependent on the experience and training of the observer. The assessment is both time-consuming and labor-intensive [8]. Moreover, inter-observer variability in VI-IIFT is well documented and represents a significant challenge for standardization in routine diagnostics, as highlighted by Rigon et al. [8,9]. These challenges pose limitations for standardized, high-throughput diagnostic workflows and may delay or complicate the classification process in clinical practice. As a result, increasing attention has turned toward the development of automated interpretation systems based on artificial intelligence (AI) pattern recognition. Mounting evidence supports the usefulness of convolutional neural network (CNN)-based machine learning tools for ANA fluorescence intensity detection and classification [10,11]. These technologies aim to provide standardized, rapid, and reproducible analysis of fluorescence patterns while maintaining high diagnostic accuracy.

This pilot study compares classical VI-IIFT with an AI-based IIFT (AI-IIFT) interpretation system (akiron^®^ NEO), focusing on diagnostic concordance and pattern recognition in accordance with the ICAP classification.

## 2. Materials and Methods

The study was performed in the immunology laboratory of a tertiary care center affiliated with the University of Regensburg, which specializes in the management of autoimmune-mediated rheumatic diseases. All serum samples analyzed using ANA-IIFT as part of routine clinical care over the course of a one-month period (February 2024) were included.

The study was conducted according to the guidelines of the Declaration of Helsinki, and was approved by the Ethics Committee of the University of Regensburg (protocol code 15-101-0029, date of approval 11 March 2015).

### 2.1. Routine ANA Reading

Classical ANA-IIFT was performed using an assay with HEp-2 cell-coated slides from AESKU (Aesku.Diagnostics GmbH & Co. KG, 55234 Wendelsheim, Germany) according to the manufacturer’s protocol with VI conducted via a Zeiss LED fluorescence microscope (Zeiss, Oberkochen, Germany). Serum samples were initially tested at a dilution of 1:80. If either visual or automated interpretation yielded a positive result, further serial dilutions (1:160, 1:320, 1:640, 1:1280, 1:2560, 1:5120, 1:10,240, and 1:20,480) were performed until a negative result was reached, or the highest dilution (1:20,480) remained positive. ANA-IIFT without detectable fluorescence was classified as negative, while those showing a defined staining pattern were considered positive. Positive patterns were classified for the predominant fluorescence pattern observed at the highest positive dilution based on the ICAP.

### 2.2. Automated AI-Aided ANA Interpretation

In parallel, all samples underwent a second ANA-IIF assay using HEp-2 cell-coated slides from Medipan (15827, Blankenfelde-Mahlow, Germany) in accordance with the manufacturer’s instructions [12]. Fluorescent images were read automatically and classified using an AI-based software with the akiron^®^ NEO system (Medipan). The akiron^®^ NEO is a benchtop Immunofluorescence Assay (IFA) analyzer for automated digital imaging of processed immunofluorescence slides to support the serological diagnosis of autoimmune diseases. The akiron^®^ NEO software (version 1.0.0) enables objective positive/negative classification of ANA HEp-2 assay results using 20x magnification and delivers automated pattern recognition according to the Competent Level of the ICAP classification. In addition, the software provides endpoint titer determination based on the quantification of image fluorescence intensity [13].

HEp-2 cells that were classified as positive by automated IIFT interpretation were re-evaluated by two human experts through visual interpretation, to assess inter-observer agreement.

### 2.3. Control for Substrate-Related Variability

Due to the use of HEp-2 cell substrates from different manufacturers (AESKU for expert visual interpretation and Medipan for the automated system), an additional comparison was performed based on 71 of the 73 AI-positive samples for which digital pattern images were available. In this analysis, both AI-assisted interpretation and visual assessment by two independent experts were applied to the same HEp-2 cell assay (Medipan) to allow for a standardized comparison.

### 2.4. Statistical Analysis

Cohen’s kappa (κ) was determined as the coefficient of agreement to evaluate the concordance between two classifications based on nominal or ordinal scales, using MedCalc Statistical Software version 19.2.1. (MedCalc Software Ltd., Ostend, Belgium).

## 3. Results

Of the 143 samples analyzed, both methods, VI- and AI-IIFT, classified 48 samples (34%) as ANA negative (Table 1). Of the 49 samples classified as negative by classical VI-IIFT, 48 (98%) were also detected as negative by automated AI-based IIFT. Among the 94 samples with a titer determined by VI-IIFT, 73 (78%) also demonstrated a titer by automated AI-based IIFT.

When the laboratory’s recommended cut-off of <1:80 was applied for positive–negative differentiation, the agreement between both ANA detection approaches was good (κ = 0.69, 95% CI 0.57 to 0.80) (Table 1). However, the two methods showed a statistically significant difference, with VI-IIFT demonstrating a higher rate of positive results compared to AI-IIFT. (McNemar test, 13.99%, 95% CI 7.98% to 19.99%, *p* < 0.0001). When a cut-off of ≤1/80 was used, κ improved to 0.75 (95% CI 0.64 to 0.86) (Table 2). At this cut-off, the difference between both methods was not significant (McNemar test, difference 0.00% (95% CI −5.82% to 5.82%, *p* = 1.0000).

Furthermore, we analyzed the correlation between titer levels determined by VI-IIFT and those obtained through automated AI-IIFT. As shown in Table 3, the AI-IIFT tended to classify more samples as negative, particularly those with low titers as determined by VI-IIFT. Overall, lower titer levels (e.g., 1:80) were more frequently interpreted as negative by AI-IIFT, whereas higher titer levels (e.g., ≥1:1280) tended to be classified at even higher titers, with a distinct clustering of results around 1:10,240.

Accurate classification of the staining pattern is essential in the assessment of positive ANA results, as it offers key information on the underlying autoantibody specificity and supports targeted reflex testing. The classification follows the internationally recognized nomenclature established by the ICAP initiative. The Competent Level was used for the comparison of 70 samples with positive patterns in both methods (Table 4).

Of the 70 samples, 49 (70%) showed concordant classification by VI-IIFT and AI-IIF (Table 4). Agreement was moderate with a κ of 0.54 (95% CI 0.39 to 0.69). All 30 anti-cell (AC)4/5 patterns (100%) analyzed by VI-IIFT were confirmed with AI-IIFT, whereas AI-IIFT additionally classified 16 sera for the nuclear fine and coarse speckled pattern. More than half of the discordant results (9/16, 56%) were due to an AC2 classification (dense fine speckled) by VI-IIFT. Three of these 9 sera (33%) received an additional AC1 classification by AI-IIFT. Of note, four of the sera classified as “other” demonstrated an AC29 pattern.

For the initial analysis, different HEp-2 cell substrates were used for AI-assisted (Medipan) and visual (AESKU) interpretation. To address this limitation, we conducted an additional analysis based on 71 of the 73 AI-positive samples, for which digital pattern images were available. Under these standardized conditions, expert visual interpretation by two experienced readers was compared with the AI-assisted classification provided by the akiron^®^ NEO software (Table 5).

The experts defined 66 samples (93%) of 71 with positive patterns. The ANA pattern agreement of the 66 samples was good with a κ of 0.67 (95% CI 0.50 to 0.84). The largest pattern group was ANA-Pattern Code (AC) AC4/5, which showed 41 concordant results. Visual interpretation by two experts assigned an additional six sera to the AC-4/5 pattern. These sera were interpreted by AI-aided classification as AC1 (n = 2) or AC2 (n = 4). In contrast, the AI-based ANA pattern interpretation identified three additional AC-4/5 patterns that were classified by visual interpretation as AC-2, AC-6/7, and AC-7/8/9. When neglecting the small difference between AC2 and AC4/5, we assessed a good agreement with κ of 0.80 (95% CI 0.65 to 0.94).

## 4. Discussion

Automated image acquisition and computer-assisted pattern recognition have been extensively implemented across multiple domains of medical diagnostics, including radiology (e.g., detection of pulmonary nodules in chest computed tomography scans [14], dermatology (e.g., differentiation of benign and malignant skin lesions [15]), and histopathology (e.g., identification of architectural and cellular abnormalities in tissue sections [16]).

Building on these advances, the use of artificial intelligence for ANA pattern recognition offers potentially a logical and increasingly standardized strategy in autoantibody diagnostics, enabling reduced manual workload and improved consistency, independent of the individual examiner.

As a step toward fully automated ANA testing, we report the first results of an AI-based screening approach for ANA assessment in a routine autoimmune laboratory and compared it with classical ANA IIFT interpretation. In the framework of the AI-aided ANA detection, all samples identified as positive by the novel system and subjected to pattern assessment would still undergo manual review to verify pattern and titer results before clinical reporting.

This pilot study assessed the diagnostic accuracy of a newly developed automated IIF interpretation system, benchmarked against manual VI as performed in routine diagnostic settings. In this setting, the algorithm, previously trained and validated by the manufacturer, was tested on an independent dataset of an immunology laboratory of a tertiary care center for the first time. As an exploratory investigation without predefined sample size calculation or clinical endpoints, its overarching aim was to evaluate the system’s potential for implementation as a screening tool in ANA diagnostics and to be used as an adjunct to VI-IIFT in routine clinical care.

In our study, the AI-aided interpretation system demonstrated a good agreement of positive/negative discrimination with classical ANA IIFT (κ 0.69), although with a significant difference according to McNemar’s statistics. The main reason was the higher number of positives by classical ANA IIFT (22%), indicating a lower sensitivity of AI-aided ANA interpretation. Using a higher cut-off of ≤1/80 for positive/negative discrimination, the titer comparison showed a trend to a very good agreement (κ 0.75) with a non-significant difference between both titer detections. Therefore, laboratories should investigate the cut-offs used for ANA titer evaluation when considering the introduction of AI-aided titer interpretation to find the optimal relation between sensitivity and specificity.

Rigon et al. reported a multicentric study involving three laboratories and 556 consecutive samples, demonstrating good inter-observer agreement between visual interpretations by human experts for both fluorescence intensity and staining pattern recognition (κ = 0.60 and κ = 0.63) [9].

This is consistent with our results demonstrating kappa values ranging from 0.69 to 0.76 for positive/negative differentiation at different cut-offs. This is encouraging, especially in light of previous deep-learning studies that reported lower agreement values [17]. However, due to the inverse relationship between sensitivity and specificity, overall agreement should be interpreted with caution and supported by individual performance metrics.

In ANA diagnostics, however, the task extends beyond the binary distinction between positive and negative results. It also involves accurate determination of antibody titers and the classification of staining patterns, both of which are clinically relevant.

In our study, we observed that titer levels in the automated evaluations were frequently reported as higher than those determined by classical ANA IIFT with VI. However, we do not expect this to lead to unnecessary testing, as all positive samples above the cut-off value are routinely subjected to reflex testing after the initial IIFT screening, regardless of the reported titer. Moreover, since the current classification criteria for SARD rely on qualitative rather than quantitative ANA results, we consider the risk of overdiagnosis to be low. Nevertheless, we acknowledge that higher ANA titers may increase the diagnostic weight of the finding in the differential diagnosis of SARD and thereby prompt greater vigilance among rheumatologists.

Compared to earlier investigations, such as the 2016 study conducted in our department, our findings indicate that AI-based pattern recognition has substantially improved in its ability to differentiate ANA patterns [18]. However, the algorithm still exhibited limitations in distinguishing between closely related nuclear patterns, particularly AC-4/5 (fine-speckled) and AC-2 (dense fine-speckled).

In contrast to the study of Durmuş et al., which included a larger number of cases but used a curated image set with clearly defined patterns, our study is based on routine clinical samples and thus more reflective of real-world conditions. Despite the differences in study design, the results are comparable in terms of agreement metrics. [19]

When we compared our established ANA IIFT with the akiron^®^ NEO system, the agreement between both approaches for ANA pattern recognition according to the Competent Level of the ICAP classification was moderate. However, discrepant results could also be due to the use of different HEp-2 cell ANA assays. Therefore, we compared the ANA pattern classification of the akiron^®^ NEO system with VI by two experts using the Medipan HEp-2 cell assay. This direct comparison revealed a good agreement with a κ of 0.67. When we neglected the differences between AC2 and AC4/5, the agreement was even very good (κ 0.80).

This study has several limitations. Most importantly, the relatively small sample size limits statistical power, especially for subgroup analyses of rare ANA patterns. Additionally, different HEp-2 cell substrates (AESKU for visual, Medipan for AI interpretation) were used, which may have introduced variability due to differences in cell preparation, morphology, fixation, and fluorescence intensity. These discrepancies could affect pattern and titer classification and must be considered when interpreting the results.

To address this, future studies should use identical HEp-2 substrates for both methods and include larger, more diverse cohorts to improve generalizability. A follow-up study is currently underway in our department, using only Medipan slides and a significantly larger patient sample.

Another limitation is the lack of final clinical diagnoses. However, the data were derived from unselected, consecutive samples in routine care—unlike most AI training sets, which often use predefined, well-characterized cases. Therefore, this real-world approach offers valuable insights into the performance of AI-based interpretation under routine clinical conditions.

Based on our findings, a novel AI algorithm have been developed to enable the analysis of HEp-2 cell preparations used in routine clinical diagnostics (AESKU, Germany).

## 5. Conclusions

In conclusion, this pilot study aimed to evaluate the potential of an AI-based approach as a screening tool for ANA IIFT and yielded promising results. However, based on our findings, the algorithm requires further optimization—particularly with regard to increasing sensitivity—while maintaining the highest possible specificity, in order to ensure suitability for use in clinical screening settings.

Despite these improvements, AI-based interpretation cannot replace human expertise. Rather, it should be regarded as an additive tool, ideally suited for standardized, high-throughput screening in clinical practice—particularly in settings with low pre-test probability, where clearly negative samples could be reliably excluded without the need for further manual review. In cases with ambiguous or borderline results, however, the final interpretation should always remain with an experienced investigator, whose clinical and technical judgment is essential for ensuring diagnostic accuracy.

In addition, improvements are needed in the algorithm’s ability to estimate antibody titers accurately and to reliably distinguish between morphologically similar staining patterns. These refinements will be essential to enable safe and effective integration of AI into routine ANA diagnostics.

## Figures and Tables

**Table 1:** 2 × 2 contingency table of antinuclear antibody (ANA)-titer comparison by visual (VI) versus automated AI-based interpretation of ANA indirect immunofluorescence testing (IIFT) of 143 serum samples using a cut-off of <1/80 (negative) for both methods. Concordant results are shown in bold.

Classical VI-IIFT, n (%)	AI-Based IIFT		
	Negative	Positive	Total
Negative	**48** (98)	1 (2)	49 (100)
Positive	21 (22)	**73** (78)	94 (100)
Total	69 (48)	74 (52)	**143** (100)

**Table 2:** 2 × 2 contingency table of antinuclear antibody (ANA) titer comparison by visual (VI) versus automated AI-based interpretation of ANA indirect immunofluorescence testing (IIFT) of 143 serum samples using a cut-off of ≤1/80 (negative) for both methods. Concordant results are shown in bold.

Classical VI-IIFT, n (%)	AI-Based IIFT		
	≤1/80	>1/80	Total
≤1/80	**61** (87)	9 (13)	70 (100)
>1/80	9 (12)	**64** (88)	73 (100)
Total	70 (49)	73 (51)	**143** (100)

**Table 3:** Visual indirect immunofluorescence testing (VI-IIFT) versus automated AI-based indirect immunofluorescence testing (AI-IIFT) detection of antinuclear antibody (ANA) titers of 143 serum samples by indirect immunofluorescence. The concordant results are shown in bold with a grey background. NEG: negative.

							AI-IIFT				
VI-IIFT	NEG	1:80	1:160	1:320	1:640	1:1280	1:2560	1:5120	1:10,240	1:20,480	Total
NEG	**48**		1								49
1:80	12	**1**		3	3	1			1		21
1:160	4		**8**	6							18
1:320	2			**1**	2	3	2	1	2		13
1:640	3				**2**	1	2		8		16
1:1280				1	2	**0**	1		10		14
1:2560					1		**0**	1	5		7
1:5120								**0**	5		5
1:10,240									**0**		0
1:20,480										**0**	0
total	69	1	9	11	10	5	5	2	31	0	**143**

**Table 4:** Comparison of antinuclear antibody (ANA) patterns by visual (classical) versus automated AI-based interpretation of ANA indirect immunofluorescence testing (IIFT) of 70 samples demonstrating positive patterns in both methods. Concordant results are highlighted in bold with a grey background.

Visual Interpretation	AI Interpretation							
	AC1	AC2	AC3	AC4/5	AC6/7	AC8/9/10	Other	Total
AC1	**4**	2		1				7
AC2		**5**		9 ***				14
AC3			**8**	1				9
AC4/5				**30**				30
AC6/7				1				1
AC8/9/10				3 ****		**2**		5
other	1 **	1 *		1 **		1 **		4

* AC4 + AC29 by visual interpretation, ** AC29 by visual interpretation, *** three sera with additional AC1 by AI-based interpretation, **** two sera with additional AC1 by AI-based interpretation.

**Table 5:** Comparison of antinuclear antibody (ANA) patterns by visual versus automated AI-based interpretation of ANA indirect immunofluorescence testing (IIFT) of 66 samples demonstrating positive patterns in the ANA assay of Medipan used for automated ANA interpretation. Concordant results are highlighted in bold with a grey background.

Visual Interpretation	AI Interpretation							
	AC1	AC2	AC3	AC4/5	AC6/7	AC8/9/10	Other	Total
AC1	**1**							1
AC2	1	**3**		1				5
AC3			**7**					7
AC4/5	2 *	4		41 ***				47
AC6/7				1				1
AC8/9/10			1	1 **		**3**		5
other								

* one serum with additional AC24 by visual interpretation, ** AC4 + AC1 by AI-based interpretation, *** three sera with additional AC1 by AI-based interpreatation.

## Data Availability

Data are not publicly available.

## Abbreviations

The following abbreviations are used in this manuscript:

AC	Anti-Cell
AI	Artificial Intelligence
AI-IIFT	Indirect Immunofluorescence Testing Using Artificial Interpretation
ANA	Antinuclear Antibodies
CNN	Convolutional Neuronal Network
EULAR	European League Against Rheumatism
HEp-2	Human Epithelial-2
ICAP	International Consensus on ANA Patterns
IFA	Immunofluorescence Assay
IgG	Immunglobulin G
SARD	Systemic Autoimmune Rheumatic Diseases
SLE	Systemic Lupus Erythematosus
IIFT	Indirect Immunofluorescence Testing
VI	Visual Interpretation
VI-IIFT	Indirect Immunofluorescence Testing Using Visual Interpretation
